# Processes and pathways to binge eating: development of an integrated cognitive and behavioural model of binge eating

**DOI:** 10.1186/s40337-019-0248-0

**Published:** 2019-06-07

**Authors:** Amy L. Burton, Maree J. Abbott

**Affiliations:** School of Psychology, The University of Sydney, Camperdown, NSW Australia

**Keywords:** Binge eating, Model, Cognitive Behavioural, Metacogntive, Eating disorder, Structural equations modelling

## Abstract

**Background:**

There are a number of factors commonly believed to be important to the development and maintenance of binge eating that have been identified across multiple models and theories in the psychological literature. In the present study, we sought to develop and test a psychological model for binge eating that incorporated the main variables identified in the literature to drive binge eating behaviour; specifically, core low self-esteem, negative affect, difficulty with emotional regulation, restricted eating and beliefs about eating.

**Methods:**

Questionnaire data was collected from 760 unselected participants. The proposed model of binge eating was developed, bivariate relationships between the included variables were assessed, and the goodness-of-fit of this new model was evaluated using structural equations modelling.

**Result:**

The results identified significant bivariate relationships between all the included variables. While the originally proposed model did not provide a good fit to the data, the revised version of the model provided a good fit to the data.

**Conclusions:**

Supporting, integrating and building upon the current existing psychological models of binge eating, this study presents a new integrated cognitive and behavioural model of binge eating. The dual-pathway to binge eating identified in the new model provides a different way to understand transdiagnostic binge eating.

## Plain English summary

This paper describes the development and assessment of a new way to understand the behaviour of binge eating. Previous research has identified a number of factors that appear to lead to the development of binge eating and have been found to contribute to maintaining binge eating in those with eating disorders. The model presented in this paper considers the role of core low self-esteem, negative emotion (e.g. depression, anxiety and stress), difficulty with emotional regulation, restricted eating (e.g. dieting) and particular unhelpful beliefs about eating. The results of this study provide support for the relationship between binge eating and the included factors (core low self-esteem, negative emotion etc.). In particular, the importance of core low self-esteem for the development and maintenance of binge eating is highlighted by the results of the paper.

## Background

Binge eating involves the sense of ‘losing control’ over one’s eating and consuming a large amount of food within a short duration of time, typically accompanied by feelings of guilt, shame, disgust and depression. Binge eating is a feature of bulimia nervosa (BN), binge eating disorder (BED) and the binge-purge type of anorexia nervosa (AN-BP) [1]. Lifetime and point prevalence data has demonstrated that between 1.5 and 2.9% of people have experienced BN, as many as 2.9 to 5.6% of people have met criteria for BED, and 0.4 to 0.9% of people have experienced anorexia nervosa (AN) [2–6]. Among the general community, studies have found that up to 7.2 to 13% of the population currently engage in regular binge eating episodes and the prevalence of binge eating in the community is increasing over time [3, 7]. Binge eating is associated with obesity, a number of chronic physical and mental health conditions, poorer quality of life, and impaired social functioning [4, 5, 8–10]. Given the high prevalence and seriousness of associated comorbid conditions, a sound understanding of the causes and maintenance of binge eating is required so that effective and affordable treatments can be developed.

A number of cognitive and behavioural models of binge eating have been proposed (see Williamson, White, York-Crowe and Stewart, 2004 [11] and Burton & Abbott, 2017 [12] for a comprehensive summary of the existing models). Many of these models overlap in hypothesised constructs; reviews of existing models have identified a number of features common among the leading psychological models of binge eating [11, 12]. A number of theories and models have hypothesised that the factors of dietary restraint, negative affect, poor emotional regulation skills, low self-esteem and the presence of thoughts/beliefs about food and eating play an important role in the development and maintenance of binge eating [12].

### Dietary restraint

One of the earliest models to account for binge eating was that of the ‘dietary restraint theory’ [13, 14]. This theory was based on both clinical observation and animal studies and proposed that a combination of dieting and restrictive eating is a precipitating factor that causes people to engage in binge eating [14]. Since it was first proposed, a growing body of evidence has supported the causal link between dietary restraint and binge eating [15], and dietary restraint has been included as a precipitating factor in many of the theories and models of binge eating that have since been developed [12]. Of note, the importance of dietary restraint is emphasised in the influential cognitive-behavioural model of BN by Fairburn, Cooper, and Cooper [16], the transdiagnostic model of eating disorders [17], the dual-pathway model [18], and the functional analysis of binge eating [19]. However, the presence of dietary restraint on its own does not account for the development of all binge eating; because of this, the dietary restraint model has received criticism for being oversimplified and for not providing an explanation for the maintenance of binge eating that occurs in those who do not engage in restrained eating [19, 20].

### Negative affect and emotional regulation

Many psychological models have proposed that binge eating is preceded by the experience of negative affect in the form of distress or depression and that binge eating is used as a way to cope with or to avoid these negative emotions [12]. This idea is explored in the escape theory proposed by Heatherton and Baumeister [21], which predicts that binge eating occurs as a way for the individual to ‘escape’ from aversive emotional states. In this way, binge eating can be seen as a coping mechanism and/or a way to avoid unpleasant emotions that can be used by individuals who experience difficulty with regulating their emotional state. In addition to the escape theory, poor emotional regulation skills in combination with the experience of negative affect are hypothesised to lead to binge eating across a number of current binge eating models, including McManus and Waller’s functional analysis of binge eating [19], Fairburn and colleagues’ cognitive behavioural model [22] and the transdiagnostic model of eating disorders [17], and the cognitive model of BN [23]. Evidence from clinical observation, naturalistic and observational studies, and experimental laboratory studies has supported the link between negative affect, difficulty with emotional regulation and binge eating [24–28].

### Low self-esteem

Another reliable predictor of binge eating is the presence of low self-esteem, also described as negative self-schemas in the schema framework, and negative self-beliefs or negative core beliefs about the self in the cognitive-behavioural framework [12]. Low self-esteem is hypothesised to be related to binge eating across a number of leading binge eating models, including the cognitive-behavioural model of BN [16], the transdiagnostic model of eating disorders [17], the cognitive model of BN [23], the functional analysis of binge eating [19], the escape theory [21], and the schema model of binge eating [29]. Research investigating the relationship between the presence and strength of negative self-schemas and eating disordered behaviours have consistently found that individuals with BED or BN have a higher level of negative self-schemas compared to community controls [29–31].

### Thoughts and beliefs about Food & Eating

In addition to negative self-beliefs, poor emotional regulation and negative affect, the cognitive model of BN developed by Cooper et al. [23] emphasises the role of specific beliefs about eating in the maintenance of binge eating. These specific eating beliefs have been categorised into three sets of beliefs:

1. Positive beliefs about eating; beliefs related to the role of eating in self-soothing, e.g., “eating makes me feel better”
2. Negative beliefs about eating; beliefs related to the negative consequences of eating, e.g., “I’ll get fat if I eat”
3. Permissive thoughts; thoughts that allow the person to engage in the binge episode, e.g., “it’s okay to eat when I feel stressed”, or thoughts related to the loss of control, e.g., “I can’t control my eating” (‘no control’ beliefs)

Cooper et al. [23] hypothesised that these eating beliefs are triggered by the experience of negative affect and that the positive, negative, and permissive beliefs interact and ultimately lead to a binge eating episode. Bergin and Wade [32] used multiple regression analyses and structural equation modelling (SEM) to test the predictions of the cognitive model of BN. Results of these analyses identified an association between negative self-beliefs and negative affect, an association between negative affect and eating beliefs, and an association between both positive and permissive beliefs and binge eating. There was no association between the negative beliefs and binge eating, although an association between negative beliefs and compensatory behaviours was identified.

The most influential psychological models of binge eating differ from each other in a number of significant ways, yet they share a number of predictive variables that have demonstrated associations with binge eating. In this paper, we draw upon the evidence-based literature to formulate a new model of the maintaining factors of binge eating based on the key overlapping constructs from existing conceptualisations of binge eating.

### Hypothesised model

The authors developed a model of binge eating that focused on five variables of interest which were believed to maintain binge eating [12]. These variables were based on a review of the relevant literature which found that these following five main variables were commonly agreed to be important predisposing, precipitating and perpetuating/maintaining factors of binge eating psychopathology:

1. core low self-esteem/negative beliefs about the self.
2. the presence of negative affect/distress.
3. poor emotional regulation.
4. dietary restraint/restriction.
5. beliefs about eating, or ‘eating beliefs’.

**Fig. 1:**
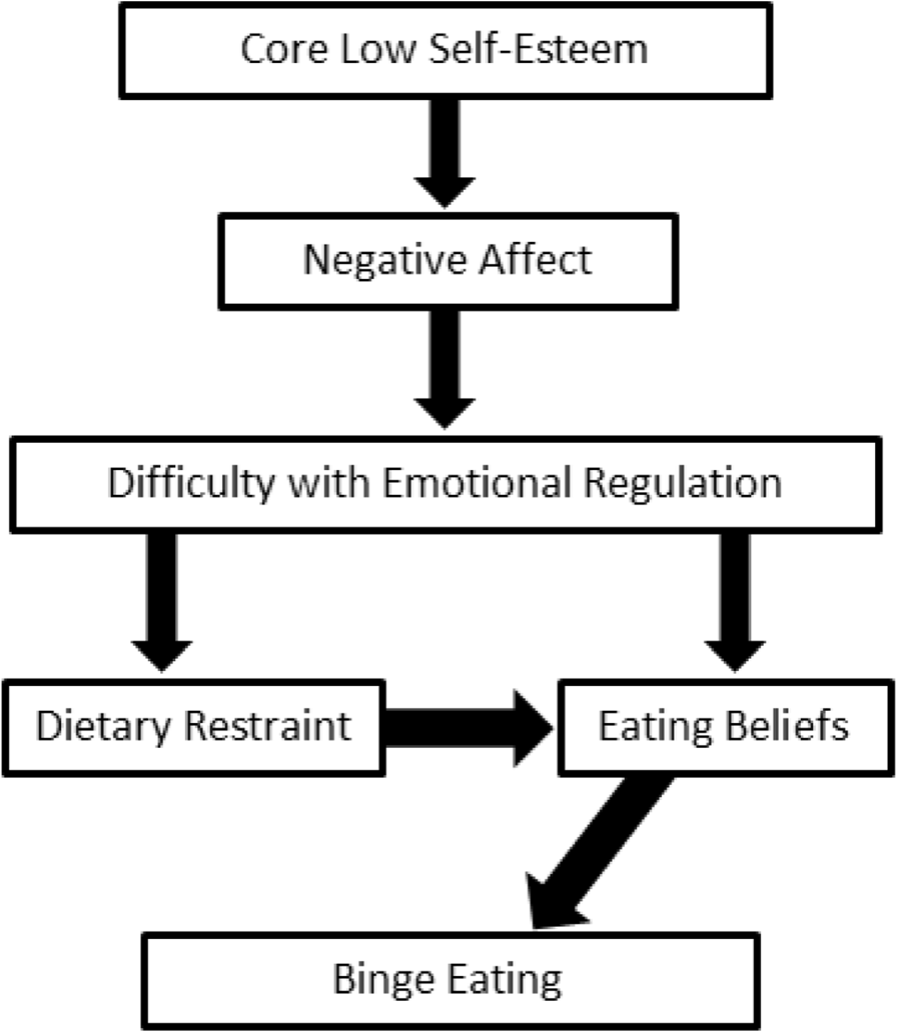
Proposed Model of Binge Eating

In the hypothesised model, individuals who have negative core beliefs about the self, or *core low self-esteem*, are predisposed to engage in binge eating (vulnerability factor). When the core beliefs are triggered, *negative affect* (low mood, anxiety, and/or stress) is experienced. Individuals who experience *difficulty with emotional regulation* feel intolerant of such negative affect and wish to find a way to neutralise the emotion. This discomfort with the negative affect is addressed by engaging in *dietary restraint* (which serves to distract from or control the emotion) and/or experiencing thoughts about food and eating (*eating beliefs*), such as positive beliefs about eating (“eating helps to control my emotions”), negative beliefs about eating (“I can’t control my eating because am weak”), and permissive beliefs about eating (“I deserve to have a pleasure like binge eating”). It is hypothesised that when these eating beliefs are triggered, binge eating occurs. The hypothesised model is presented in Fig. 1.

### Aims and hypotheses

The aim of this study was to develop and test a psychological model for binge eating that incorporated the main common variables identified across a number of existing cognitive and behavioural theories of binge eating. Based on the literature, we predicted that the variables of core low self-esteem, negative affect, difficulty with emotional regulation, dietary restraint, and beliefs about eating would be positively correlated with the behavioural symptom of binge eating. Additionally, we hypothesised that a model based on these five variables would provide an acceptable fit to the data.

## Methods

### Participants

Participants were recruited from a sample of first year psychology students at the University of Sydney. A total of 767 students participated in the study (71.2% female, mean age = 19.37 years, SD = 3.46 years, mean BMI = 21.99, SD = 3.52). For analyses performed in this study, a data set with no missing data was required. Consequently, the data from 7 participants who had missing data was removed from further analysis. Therefore, the complete data for 760 participants was used for the analyses reported (*n* = 760, 71.1% female, mean age = 19.37, SD = 3.47, mean BMI = 21.99, SD = 3.53). Of these 760 participants, 62% self-reported that they had never suffered from or been treated for a psychological condition such as depression, anxiety, psychosis, or an eating disorder. Lifetime prevalence rates of eating disorders amongst the participant sample closely resembled previously reported Australian prevalence rates (Wade et al., 2006); 2.8% of participants reported that they had suffered from or been treated for anorexia nervosa, 2.9% for bulimia nervosa, 3% for binge eating disorder, and 2.1% for an atypical eating disorder (e.g., EDNOS or OSFED). Of the 760 participants, 22.1% (*n* = 168) self-reported that they had experienced at least four binge eating episodes that were paired with a sense of loss of control over the previous 28 days, with the total sample reporting an average of 2.43 (SD = 5.09) binge eating episodes with loss of control over the previous 28 days (Range = 0 to 50). Informed consent was obtained from all individual participants included in the study.

### Procedure

Participants were asked to provide demographic information (age, gender, height, weight, previous or current mental health diagnoses) and to complete a series of questionnaires online that focused on eating behaviours and their beliefs about eating using the Qualtrics online questionnaire program.

### Measures

#### Eating disorder symptoms: dietary restraint and binge eating

The Eating Disorder Examination Questionnaire (EDE-Q) [33] version 6.0 was used to collect information on possible eating disorder symptoms and behaviours such as binge eating and restrictive eating practices. The EDE-Q is a self-report questionnaire that asks respondents to record information regarding the frequency and severity of eating and body image-related concerns and behaviours experienced over the past 28 days. The EDE-Q is a valid and reliable measure with demonstrated psychometric properties [34]. A composite of two items was used in this study as the measure of ‘binge eating’: item 14, which assesses the number of occasions over the past 28 days that the respondent has eaten “what other people would regard as an unusually large amount of food (given the circumstances)” and experienced “a sense of having lost control over your eating (at the time)”; and item 15, which assesses the number of days over the past 28 days that “such episodes of over eating occurred (i.e., you have eaten an unusually large amount of food and had a sense of loss of control at the time)”. This composite score gives an overall assessment of the severity of binge eating, with higher scores indicating increased severity of binge eating. A composite score was chosen to measure the construct of binge eating rather than a single item as the use of multiple items to measure a constructs reduces the effect of item-specific measurement error and therefore leads to more accurate research findings [35]. The restraint subscale was used as the measure of ‘dietary restraint’ in this study. The restraint subscale contains 5 items that assess the presence of fasting, dieting, calorie restriction and strict rules about food and eating. In the present study, the EDE-Q global score had a Cronbach’s alpha (α) of .94, and α = .80 for the restraint subscale.

#### Negative affect

The Depression Anxiety Stress Scales Short Form (DASS-21) [36] was included to measure the negative affect currently experienced by respondents. The DASS-21 is a self-report questionnaire used to assess depression, anxiety, and stress symptoms that have been experienced over the past week. The DASS-21 is a valid and reliable self-report instrument [37]. A total score based on all 21 items was used as the measure of ‘negative affect’ in this study. In the present study, the DASS-21 total score had a Cronbach’s alpha of .93.

#### Core low self-esteem

The Eating Disorders Core Beliefs Questionnaire (ED-CBQ) [38] was used to assess the presence of core beliefs about the self relevant to eating disorders. In the development paper, the ED-CBQ demonstrated contruct validity and internal consistency [38]. Items on the self-loathing subscale, such as “I am repulsive”, were used to assess core low self-esteem. In the present study, the Cronbach’s alpha of the ED-CBQ total was α = .92, and α = .93 for the self-loathing subscale.

#### Poor emotional regulation

The Difficulty in Emotion Regulation Scale (DERS) [39] is a 36-item self-report questionnaire used to assess difficulties with emotion regulation. The DERS assesses different aspects of emotional regulation including non-acceptance of emotional responses, difficulty engaging in goal-directed behaviour, impulse control difficulties, lack of emotional awareness, limited access to emotional regulation strategies, and lack of emotional clarity; higher scores reflect poorer emotion regulation. The DERS has demonstrated good internal consistency, construct and predictive validity and test-retest reliability [39]. The total score of the DERS was used as a measure of difficulty with emotional regulation in this study. The DERS total score demonstrated Cronbach’s alpha of .89 in the present study.

#### Eating beliefs

The Eating Beliefs Questionnaire (EBQ-18) [40] is an 18-item self-report measure that assesses three types of beliefs about food and non-hungry eating: positive beliefs such as “eating helps me cope with negative feelings”, ‘no control’ beliefs such as “once I start eating, I can’t stop”, and permissive beliefs such as “I deserve to have a pleasure like binge eating”. The EBQ-18 is a valid and reliable measure with demonstrated psychometric properties [40, 41]. The total score of the EBQ-18 was used as the measure of eating beliefs in this study. The EBQ-18 total score demonstrated a Cronbach’s alpha of .92 in the present study.

### Statistical analyses

The SPSS v22 program was used to generate descriptive statistics such as means, standard deviations and Cronbach’s alphas. Structural equations modelling was conducted using the AMOS version 22 program [42]. The overall fit of the models was assessed using a number of known indicators of goodness-of-fit; the goodness-of-fit index (GFI), the comparative fit index (CFI), the Tucker-Lewis index (TLI), the incremental fit index (IFI), the root mean square error of approximation (RMSEA), and the *p* of close fit statistic (PCLOSE). According to Hu and Bentler [43], values over .95 on the CFI, TLI, and IFI are indicative of an acceptable model fit, and RMSEA values of less than 0.06 indicate an acceptable fit. A non-significant PCLOSE statistic (>.05) indicates a ‘close’ fit of the model to the data [44].

## Results

The means and standard deviations for the included variables, and the bivariate relationships among the included variables, are presented in Table 1, with all correlations significant at *p* < .05.

**Table 1:** Descriptive Statistics and Bivariate Relationships for all Included Variables

	Mean	SD	1	2	3	4	5
1. Core Low Self-Esteem	18.47	10.84					
2. Negative Affect	36.90	11.02	.55*				
3. Difficulty with Emotional Regulation	88.38	23.43	.55*	.71*			
4. Dietary Restraint	2.23	1.29	.23*	.30*	.30*		
5. Eating Beliefs	38.72	12.55	.41*	.39*	.49*	.16*	
6. Binge Eating	5.09	9.23	.30*	.29*	.30*	.27*	.46*

*= *p* < .05

### Structural equations modelling

The hypothesised model (Model 1; see Fig. 1) was fitted to the data using the AMOS program. The regression weights for the hypothesised model are shown in Table 2; the pathway between ‘Dietary Restraint’ and ‘Eating Beliefs’ was not significant (*p* = .60). However, all other pathways described in Table 2 were significant. This model did not demonstrate acceptable fit across a number of the goodness-of-fit indices (see Table 3).

**Table 2:** Unstandardised and Standardised Regression Weights for Hypothesised Model (Model 1) (Standard Errors in Brackets)

Pathway	Unstandardised Estimate	Standardised Estimate	*p*
Core Low Self-Esteem → Negative Affect	.55 (.03)	.55	<.001
Negative Affect → Difficulty with Emotional Regulation	1.51 (.05)	.71	<.001
Difficulty with Emotional Regulation → Dietary Restraint	.02 (.002)	.30	<.001
Difficulty with Emotional Regulation → Eating Beliefs	.262 (.02)	.49	<.001
Dietary Restraint → Eating Beliefs	.168 (.32)	.017	.60
Eating Beliefs → Binge Eating	.34 (.02)	.46	<.001

**Table 3:** Goodness-of-Fit Indices for Models

Model	χ^2^/df	GFI	CFI	TLI	IFI	RMSEA (90% CI)	PCLOSE
1	17.363	0.94	0.895	0.826	0.896	.147 (.127 to .167)	.000
2	13.08	0.956	0.923	0.871	0.923	.126 (.106 to .147)	.000
3	7.612	0.975	0.962	0.93	0.963	.093 (.072 to .116)	.001
4	4.716	0.986	0.982	0.96	0.982	.070 (.047 to .095)	.074
5	2.781	0.993	0.992	0.981	0.992	.048 (.022 to .077)	.487

Inspection of the regression weights, covariances, and correlations, as well as a discussion of the theoretical meaning of the pathways in the model between the authors, led to a series of changes to the model for the purpose of improving the fit. The authors removed the non-significant pathway from ‘Dietary Restraint’ to ‘Eating Beliefs’ and a pathway from ‘Dietary Restraint’ to ‘Binge Eating’ was added (Model 2); this revised model demonstrated improved fit across the indices (see Table 3).

**Fig. 2:**
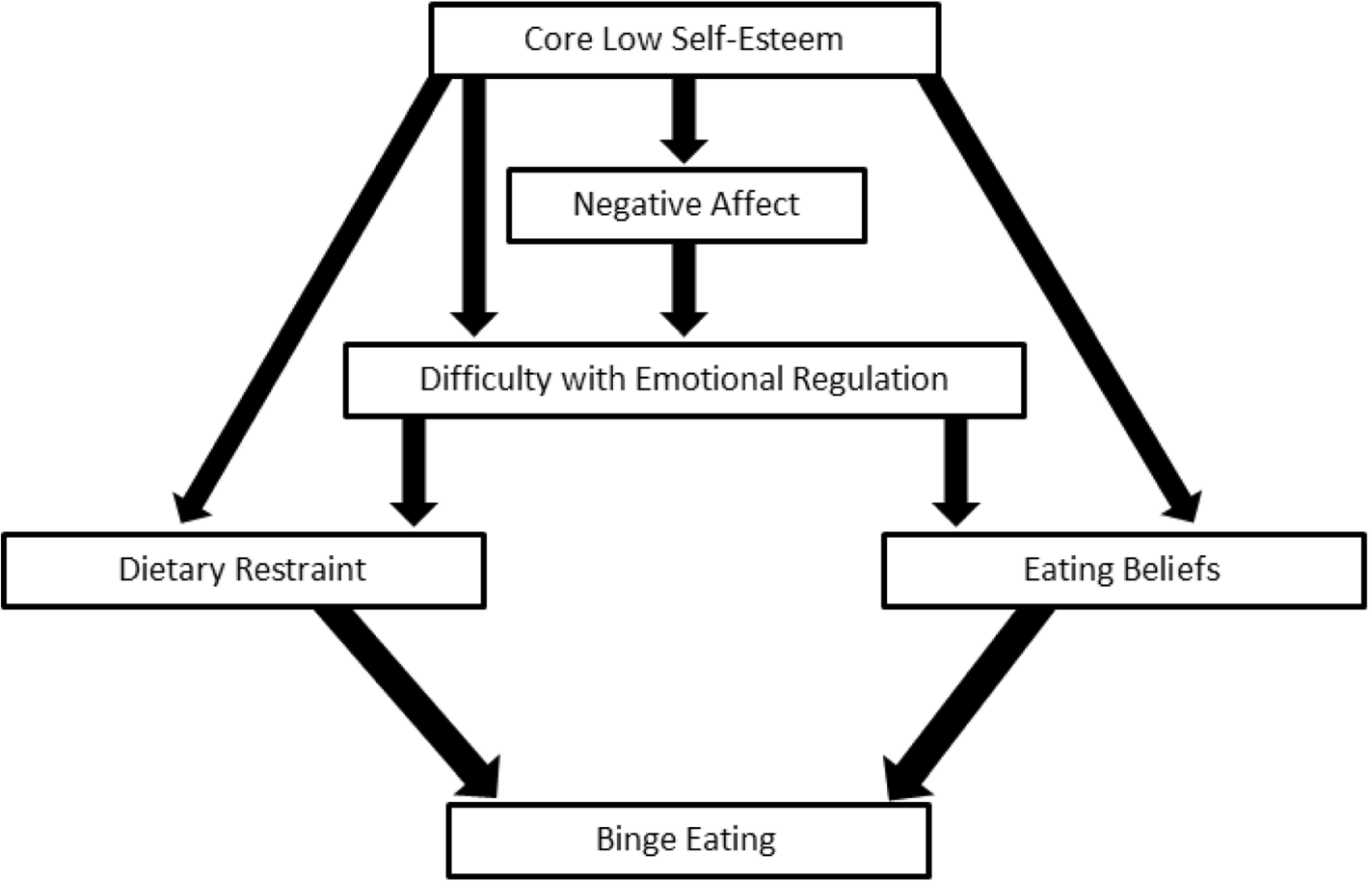
Integrated Cognitive and Behavioural Model of Binge Eating

Again, upon close inspection of the fit statistics, covariances, correlations, and discussion of the theoretical value of each pathway, three more changes were made, and each revision resulted in an improvement of fit: first, a path from ‘Core Low Self-Esteem’ to ‘Difficulty with Emotional Regulation’ was added (Model 3); then a path from ‘Core Low Self-Esteem’ to ‘Eating Beliefs’ was also included (Model 4). Model 4 demonstrated acceptable fit across most of the goodness-of-fit indices, however, the χ^2^/df value and the RMSEA value were higher than optimal. Finally, a path from ‘Core Low Self-Esteem’ to ‘Dietary Restraint’ was added (Model 5). The final model (Model 5) demonstrated good fit to the data across all the goodness-of-fit indicators (see Table 3). The regression weights for the final model are presented in Table 4 (all significant) and the pathways of the final model as shown in Fig. 2.

**Table 4:** Unstandardised and Standardised Regression Weights for Final Model (Model 5) (Standard Errors in Brackets)

Pathway	Unstandardised Estimate	Standardised Estimate	*p*
Core Low Self-Esteem → Negative Affect	.55 (.03)	.54	<.001
Core Low Self-Esteem → Difficulty with Emotional Regulation	.49 (.06)	.23	<.001
Core Low Self-Esteem → Dietary Restraint	.02 (.01)	.17	<.001
Core Low Self-Esteem → Eating Beliefs	.23 (.04)	.20	<.001
Negative Affect → Difficulty with Emotional Regulation	1.25(.06)	.59	<.001
Difficulty with Emotional Regulation → Dietary Restraint	.01 (.002)	.20	<.001
Difficulty with Emotional Regulation → Eating Beliefs	.207 (.02)	.39	<.001
Dietary Restraint → Binge Eating	1.435 (.23)	.20	<.001
Eating Beliefs → Binge Eating	.315 (.02)	.43	<.001

## Discussion

The aim of the present study was to develop and test a new psychological model of binge eating which included variables hypothesised by the leading existing cognitive and behavioural theoretical models on the maintenance of binge eating. In particular, our model bears a number of shared variables with the models of functional analysis of binge eating [19], the transdiagnostic model of eating disorders [17], and the cognitive model of BN [23].

The included variables of Core Low Self-Esteem, Negative Affect, Difficulty with Emotional Regulation, Dietary Restraint, and Eating Beliefs were all found to be positively correlated with Binge Eating. In addition, significant bivariate relationships were found amongst all the included variables. The originally hypothesised model did not demonstrate acceptable fit to the data, and the pathway between dietary restraint and eating beliefs was not found to be significant. Once this non-significant pathway was removed and a number of additional pathways between variables that had strong covariances were added, a modified model demonstrated good fit to the data. Therefore, the results indicated that the final revised model (Model 5; refer to Fig. 2) provided the best fit to the data. We present this final model as a new way to conceptualise the maintaining factors for binge eating; that is, the integrated cognitive and behavioural model of binge eating.

The results of this study provide further support for the relationship between the predicted variables (Core Low Self-Esteem, Negative Affect, Difficulty with Emotional Regulation, Dietary Restraint, and Eating Beliefs) and the outcome of binge eating. In particular, the role of Core Low Self-Esteem has been highlighted as particularly important, with the significant pathways from Core Low Self-Esteem to Binge Eating being mediated through a number of direct (via Dietary Restraint or Eating Beliefs) and indirect (via negative affect or Difficulty with Emotional Regulation) pathways. Also of interest is the strength of the bivariate relationship between Eating Beliefs and Binge Eating, as well as the strength of the relationship between Eating Beliefs and the two predicted preceding variables: Core Low Self-Esteem and Difficulty with Emotional Regulation. Of the included variables, Eating Beliefs showed the least amount of existing evidence supporting its role in the maintenance of binge eating in the literature due to its relative novelty in the field, being first proposed in 2004 whilst the other variables first appeared in the literature in between 1975 and 1991. These findings regarding Eating Beliefs (comprised of positive beliefs, permissive beliefs, and ‘no control’ beliefs) are in line with those reported in Bergin and Wade [32] who found that positive and permissive/no control thoughts predicted binge eating.

Furthermore, the results of this study provide support for the role of dietary restraint as an important predictive factor for binge eating. Of particular interest is the non-significant pathway between Dietary Restraint and Eating Beliefs indicating that these two variables act independently from one another. As a result, the final model includes a ‘dual pathway’ to binge eating; either via Dietary Restraint or via the activation of Eating Beliefs. In fact, it is possible that this ‘dual pathway’ may indicate two separate types of binge eating. The first, mediated by Dietary Restraint, more closely resembles the pathways to binge eating hypothesised in the transdiagnostic model [17]. This first pathway could represent the type of binge eating that is more strongly maintained by a sense of loss of control and may be more commonly observed in people with restrictive eating disorders such as AN-BP and certain cases of BN. The second, mediated by Eating Beliefs, more closely resembles the pathway to binge eating proposed in the cognitive model of BN [23]. This second type of binge eating could represent the type of binge eating that is more strongly maintained by its function to comfort and self-soothe, and may be more commonly observed in people who do not restrict their eating such as BED, certain cases of BN, and sub-clinical binge eating.

### Similarities to other models

The new model presented in this paper posits that core low self-esteem is a major underlying predisposing factor for binge eating. This is in line with the functional analysis of binge eating [19], the transdiagnostic model [17], the cognitive model of BN [23], and a number of other binge eating models [12] that also identify low self-esteem as an important predisposing factor for the development of binge eating. The new model proposes that when core low self-esteem is triggered (experienced as a range of feelings and beliefs, measured by negative statements about the self), negative affect is experienced (in line with the cognitive model of BN [23]). The new model then suggests that a difficulty with regulating the negative affect is experienced, and as such, the individual responds in one of two ways:

1. They engage in, or attempt to engage in, restrictive eating practices as a way to cope with the negative affect attempting to gain ‘affective’ control, and this restrained eating then triggers binge eating (as in the dietary restraint theory, functional analysis of binge eating, transdiagnostic model of eating disorders, and many others [12]).

Or

(2) Beliefs about eating are activated and themselves trigger binge eating as a means of functionally coping with negative affect (as in the cognitive model of BN [23]).

### Unique contributions

In addition to synthesising the main evidence-based variables hypothesised to lead to and maintain binge eating, the new model presented in this paper also offers some unique insights into the way in which these variables relate to one another to lead to binge eating, above and beyond what has already been demonstrated in previous studies. Most important is the dual pathway to binge eating identified in this model, indicating the possibility of two different ‘types’ of binge eating which are maintained by different processes. The relevance and necessity of dietary restraint in the development and maintenance of binge eating has been contested in the literature and amongst clinicians [19, 45]; the dual pathway presented in this new model provides an alternative in that binge eating can be triggered either by restrained eating or by the activation of particular beliefs about eating. Furthermore, the new model presented in this paper provides an integrated cognitive-behavioural model of binge eating which is transdiagnostic, and focused on behavioural symptoms rather than simply the presence or absence of a diagnosis.

### Limitations

It is important to note that the results of this study need to be interpreted in the context of a number of limitations. Firstly, the results are limited by the instruments used to measure the variables and associated constructs. For example, both binge eating and dietary restraint were measured by the same instrument, the EDE-Q, and therefore it is possible that the relationship between these two variables might have been artificially enhanced due to the fact that they were measured together. Also, the instruments used assess different time periods, for example, while the EDE-Q assesses symptoms experienced over the previous 28-days, the items in the DASS-21 refer to the past week. Therefore, in order to be able to more accurately assess if binge eating behaviours are occurring at the same time as negative affect it is recommended that future studies utilise measures referring to the same period of time and to test the model with a range of different measures for each factor. Future research should also assess the stability of the model fit if alternative questionnaires are used to measure the proposed predictive variables. This is especially important with regard to the measurement of binge eating, as the EDE-Q has received some criticism with regard to the accuracy of the measurement of binge eating [33, 34, 46]. Secondly, the participants included in this study were non-clinical, and the eating disorder symptoms (including binge eating) were based on a self-report measure. Future studies investigating the new model should test the fit of the model to a clinical sample whose binge eating status and/or eating disorder diagnosis has been assessed by a trained clinician using semi-structured interviews such as the Eating Disorders Examination [22]. Furthermore, it is important to emphasise that this paper represents a preliminary investigation of this new model, and further research is required to assess the utility and validity of this new model in longitudinal research and in intervention-based research.

## Conclusion

Based on the existing literature, a cognitive and behavioural model of transdiagnostic binge eating was developed and tested. The resultant model provides a good fit to the data and offers a novel way to conceptualise binge eating that supports, integrates, and builds upon the current existing psychological models of binge eating. The model can provide a framework for understanding the causal and maintenance factors of binge eating and provides several areas for intervention. Based on the model, treatments that target the core low self-esteem and improve emotional regulation skills are likely to lead to reductions in binge eating. Depending on whether the individual’s binge eating is usually triggered by dietary restraint or usually triggered by the activation of eating beliefs, or if their binge eating can be triggered by either of these factors, then treatment approaches can be personalised to focus more on addressing the dietary restraint, addressing unhelpful beliefs about eating, or on addressing both. Results presented here are preliminary, and further investigation is required to assess the accuracy and the clinical utility of the model for individuals seeking treatment for binge eating.

## Abbreviations

AN: Anorexia Nervosa
AN-BP: Anorexia Nervosa (binge/purge subtype)
BED: Binge Eating Disorder
BMI: Body Mass Index
BN: Bulimia Nervosa
CFI: Comparative Fit Index
CI: Confidence Intervals
DASS-21: Depression Anxiety Stress Scales
DERS: Difficulty in Emotion Regulation Scale
df: degrees of freedom
EBQ-18: Eating Beliefs Questionnaire (18 items)
ED-CBQ: Eating Disorders Core Beliefs Questionnaire
EDE-Q: Eating Disorders Examination Questionnaire
EDNOS: Eating Disorder Not Otherwise Specified
GFI: Goodness of Fit Index
IFI: Incremental Fit Index
OSFED: Other Specified Feeding or Eating Disorder
RMSEA: Root Mean Square Error of Approximation
SD: Standard Deviation
SEM: Structural Equations Modelling
SPSS: Statistical Package for Social Sciences
TLI: Tucker-Lewis Index
